# Single-cell RNA sequencing deconvolutes the *in vivo* heterogeneity of human bone marrow-derived mesenchymal stem cells

**DOI:** 10.7150/ijbs.61950

**Published:** 2021-10-11

**Authors:** Zun Wang, Xiaohua Li, Junxiao Yang, Yun Gong, Huixi Zhang, Xiang Qiu, Ying Liu, Cui Zhou, Yu Chen, Jonathan Greenbaum, Liang Cheng, Yihe Hu, Jie Xie, Xucheng Yang, Yusheng Li, Martin R. Schiller, Yiping Chen, Lijun Tan, Si-Yuan Tang, Hui Shen, Hong-Mei Xiao, Hong-Wen Deng

**Affiliations:** 1 Xiangya School of Nursing, Central South University, Changsha, 410013, China; Laboratory of Molecular and Statistical Genetics, College of Life Sciences, Human Normal University, Changsha, 410081, China.; 2 Tulane Center for Biomedical Informatics and Genomics, School of Medicine, Tulane University, New Orleans, 70112, USA.; 3 Laboratory of Molecular and Statistical Genetics, College of Life Sciences, Human Normal University, Changsha, 410081, China.; 4 School of Basic Medical Science, Central South University, Changsha, 410008, China.; 5 Center of Reproductive Health, System Biology and Data Information, Institute of Reproductive & Stem Cell Engineering, School of Basic Medical Science, Central South University, Changsha, 410008, China.; 6 Department of Orthopedics, Xiangya Hospital, Central South University, Changsha, 410008, China.; 7 Department of Orthopedics and National Clinical Research Center for Geriatric Disorders, Xiangya Hospital, Central South University, Changsha, 410008, China.; 8 Hunan Women's Research Association, Changsha, 410011, China.; 9 Nevada Institute of Personalized Medicine and School of Life Science, 4505 S. Maryland Pkwy, Las Vegas, NV 89154-4004, USA.; 10 Department of Cell and Molecular Biology, School of Science and Engineering, Tulane University, New Orleans, LA 70112, USA.

**Keywords:** single-cell RNA sequencing (scRNA-seq), mesenchymal stem cell (MSC), bone marrow, osteogenesis, chondrogenesis, adipogenesis

## Abstract

Bone marrow-derived mesenchymal stem cells (BM-MSCs) are multipotent stromal cells that have a critical role in the maintenance of skeletal tissues such as bone, cartilage, and the fat in bone marrow. In addition to providing microenvironmental support for hematopoietic processes, BM-MSCs can differentiate into various mesodermal lineages including osteoblast/osteocyte, chondrocyte, and adipocyte that are crucial for bone metabolism. While BM-MSCs have high cell-to-cell heterogeneity in gene expression, the cell subtypes that contribute to this heterogeneity *in vivo* in humans have not been characterized. To investigate the transcriptional diversity of BM-MSCs, we applied single-cell RNA sequencing (scRNA-seq) on freshly isolated CD271^+^ BM-derived mononuclear cells (BM-MNCs) from two human subjects. We successfully identified LEPR^hi^CD45^low^ BM-MSCs within the CD271^+^ BM-MNC population, and further codified the BM-MSCs into distinct subpopulations corresponding to the osteogenic, chondrogenic, and adipogenic differentiation trajectories, as well as terminal-stage quiescent cells. Biological functional annotations of the transcriptomes suggest that osteoblast precursors induce angiogenesis coupled with osteogenesis, and chondrocyte precursors have the potential to differentiate into myocytes. We also discovered transcripts for several clusters of differentiation (CD) markers that were either highly expressed (e.g., CD167b, CD91, CD130 and CD118) or absent (e.g., CD74, CD217, CD148 and CD68) in BM-MSCs, representing potential novel markers for human BM-MSC purification. This study is the first systematic *in vivo* dissection of human BM-MSCs cell subtypes at the single-cell resolution, revealing an insight into the extent of their cellular heterogeneity and roles in maintaining bone homeostasis.

## Introduction

The human bone tissue is a complex system that consists of diverse cell types including osteoblast/osteocyte, osteoclast, and chondrocyte (collectively known as “bone cells”), together with various supporting cells such as adipocyte, fibroblast, and hematopoietic cells among others. A delicate balance of bone formation/resorption is critical for maintaining bone health, and therefore bone cells must work together to maintain bone strength and mineral homeostasis. Despite the extensive study of bone cells, their underlying biology remains poorly understood. While osteoclasts are of hematopoietic origin and derived from the “monocyte/macrophage-preosteoclast-osteoclast” differentiation trajectory 1, the detailed origins of osteoblast/osteocyte and chondrocyte are not as well characterized.

Currently, cells that give rise to osteoblast/osteocyte, chondrocyte, and adipocyte are generally referred to as “mesenchymal stromal/stem cells” (MSCs), which are non-hematopoietic bone marrow stromal cells with fibroblast colony-forming unit (CFU-F) and multi-differentiation capacity 2. Typically, the human bone-marrow derived MSCs (BM-MSCs) are isolated with a combination of non-specific cell-surface markers such as high level of CD271, CD44, CD105, CD73, CD90, and low level/absence of CD45, CD34, CD14 or CD11b, CD79a or CD19, and human leukocyte antigen HLA-DR 3,4. Among these markers, CD271 shows great efficiency to sort MSCs either alone or in combination with negative selection of markers such as CD45 5,6. Additionally, LEPR (leptin receptor, or CD295) is used for isolating BM-MSCs in transgenic labeling mice 7,8. Although these cell markers are candidates for isolating BM-MSCs, recent evidence suggests that the BM-MSCs are a heterogeneous group of cells for some cell markers. For instance, Akiyama et al. 9 demonstrated that a small portion of BM-MSCs express CD45 and CD34, which are traditionally regarded as negative markers. Meanwhile, some studies also suggested that only around 50% of MSCs are positive for CD105 10,11, a cell marker previously considered universally expressed by MSCs derived from different tissue 12.

The extent of the cellular heterogeneity among the BM-MSCs is not well-defined, although a few studies have proposed some novel subtypes. One study reported a subset of cultured mouse BM-MSCs that are distinct from regular BM-MSCs based upon differential attachment to plastic culture dishes, proliferation, and self-renewal patterns 9. Another study examining cultured human BM-MSCs demonstrated that CD264 marks a subpopulation of aging human BM-MSCs with differential fibroblast colony forming efficiency 13. Several other efforts have attempted to deconvolute the heterogeneity of BM-MSCs through the identification of the differentiation trajectory associated with a given subpopulation. For example, one study found that effective chondrocyte differentiation could only be induced in human MSCA-1^+^CD56^+^ BM-MSCs, while adipocytes are derived only from MSCA-1^+^CD56^-^ BM-MSCs *in vitro* 14. Another study identified “skeletal stem cells” in both mice and humans, which give rise to bone, stroma, and cartilage cells *in vivo* in mice, but not adipocytes or myocytes 15,16.

Single-cell RNA sequencing (scRNA-seq) has recently emerged as a powerful approach to study cell heterogeneity in complex tissues. scRNA-seq measures transcriptional profiles of many cells at single-cell resolution, which can be clustered to distinguish and classify cell subtypes, infer developmental trajectories, and identify novel regulatory mechanisms 17,18. scRNA-seq technology represents a major advancement beyond the conventional bulk RNA-seq transcriptomics approach which attempts to infer biological mechanisms from average gene expression, weighted by the unknown proportions of unknown cell subtypes, across a heterogeneous cell population. Several studies have applied scRNA-seq to bone marrow stroma cells. However, these studies were either conducted in mice 7,19 or cultured cells from human subjects 20,21, which may not accurately represent the transcriptional profile of human primary BM-MSCs *in vivo* 22,23.

Our current work is the first systematic scRNA-seq analysis of freshly isolated human CD271^+^ bone marrow mononuclear cells (BM-MNCs). We have successfully identified LEPR^hi^CD45^low^ BM-MSCs in the CD271^+^ BM-MNC population and further revealed distinct subpopulations in LEPR^hi^CD45^low^ BM-MSCs along with their differentiation relationships and functional characteristics. By comparing the expression pattern of LEPR^hi^CD45^low^ BM-MSCs with CD45^hi^ hematopoietic cells, we have also identified several potential novel markers for human BM-MSC purification. Our findings provide significant insight into the identities and complexities of human BM-MSCs *in vivo*.

## Methods

### Study population

The clinical study was approved by the Medical Ethics Committee of Central South University, and written informed consents were obtained from each participant. The study population consists of two Chinese subjects who underwent hip replacement surgery at the Xiangya Hospital of Central South University in 2019, including one 84-year-old male with osteoarthritis and normal bone mineral density (BMD; BMD T-score: -0.9 at lumbar vertebrae, 2.7 at total hip) and one 67-year-old female with osteoporosis (BMD T-score: -3.3 at lumbar vertebrae, -3.7 at total hip). Study participants were screened prior to surgery based on a detailed questionnaire, medical history, and a physical examination. Subjects were excluded from the study if they had preexisting chronic conditions which may influence bone metabolism including diabetes mellitus, renal failure, liver failure, hematologic diseases, disorders of the thyroid/parathyroid, malabsorption syndrome, malignant tumors, and previous pathologic fractures 24. During hip replacement surgery, surgeons collected the bone marrow from the femoral shafts from each subject and transferred the samples to our laboratory immediately following the procedure. The samples were stored at 4 °C and processed within 24 hours after collection.

### Experimental animals

Female C57BL/6J mice were purchased from Jackson Laboratory (Bar Harbor, ME, USA). All mice were housed in pathogen-free conditions and fed with autoclaved food, and all experimental procedures were approved by the Ethics Committee of Xiangya Hospital of Central South University.

### BMD measurement

BMD (g/cm^2^) at the lumbar spine (L1-L4) and the total hip (femoral neck and trochanter) were measured with a duel energy x-ray absorptiometry (DXA) fan-beam bone densitometer (Hologic QDR 4500A, Hologic, Inc., Bedford, MA, USA). According to the World Health Organization definition 25 and the BMD reference established for Chinese populations 26, subjects with a BMD of 2.5 SDs lower than the peak mean of the same gender (T-score ≤ -2.5) were determined to be osteoporotic, while subjects with -2.5 < T-score < -1 are classified as having osteopenia and subjects with T-score > -1.0 are considered healthy.

### Human bone marrow cell dissociation

Bone marrow derived mononuclear cells (BM-MNCs) were extracted from the marrow cavity of femoral shafts using a widely applied dissociation protocol 5,6]. Briefly, the bone marrow was attenuated with PBS (1:2) and mixed gently. The mixture was then equally layered onto equal volume of Ficoll (GE health care, Chicago, IL, USA), and the buffy coat was isolated by centrifugation (440 g, 35 min, 4 °C). The separated buffy coat was transferred into a new 15 ml centrifuge tube and washed with PBS. After discarding the supernatant, red blood cells were lysed with RBC Lysis Buffer (Thermo Fisher, Waltham, MA, USA). After washing twice with PBS, the remaining MNCs were further purified with CD271 magnetic MicroBeads (Miltenyi Biotec, Bergisch Gladbach, Germany) for positive selection 6.

### Positive selection of human CD271^+^ BM-MNC

BM-MNCs were incubated for 10 min at 4-8 °C with monoclonal antibody (mAb) against CD271. After washing, the cells were incubated with anti-IgG1 immunomagnetic beads for 15 min at 4 °C. The cell suspension was placed on a column in a cell separator (Miltenyi Biotec), and the positive fraction was subjected to a second separation step. The cells were then counted and assessed for viability with a Countstar® Rigel S3 fluorescence cell analyzer (ALIT Life Science Co., Ltd, Shanghai, China).

### Isolation of murine BM-MSCs

Cells were isolated from flushed bone marrow from female C57BL/6 mice (8 weeks) and dissociated using 21G needle. Cells were then plated in 75-cm^2^ cell culture flasks containing 10 mL of MesenCult^TM^ basal expansion medium with 10× Supplement (Stemcell, Vancouver, Canada), 100 U/mL penicillin-streptomycin, L-glutamine 2 mM, and incubate at 37 °C 5% CO_2_ for one week. 0.1% MesenPure^TM^ (Stemcell) was added for the depletion of CD45^+^ cells. Stromal cells were allowed to reach 80%-90% confluency before passage or planting.

### Flow cytometry

Cells were resuspended in 100 μL of staining medium, followed by staining with fluorochrome-conjugated antibodies on ice for 20 minutes. The antibodies used in this study to identify MSCs were anti-CD45-FITC (eBioscience, clone 30-F11, 0.5 µg/test), anti-Ter119-FITC (eBioscience, clone TER-119, 0.25 µg/test), anti-CD31-FITC (eBioscience, clone 390, 1 µg/test), and anti-CD56-PE (R&D Systems, clone # 809220, 0.5 µg/test). Cells were analyzed on a Sony MA900 Cell Sorter, where CD45/Ter119/CD31^-^ cells were identified as BM-MSCs, and CD56 was used to separate CD56^+^ and CD56^-^ cell subtypes.

### Bone sectioning, immunostaining, and confocal imaging

Freshly dissected bones were fixed in 4% paraformaldehyde overnight, followed by decalcification in 10% EDTA for 1 week, and then dehydrated using a series of graded ethanol and embedded in paraffin. Samples were then cut into 5-µm-thick longitudinally oriented sections, deparaffinized in xylene, and rehydrated in decreasing concentrations of ethanol followed by distilled water. After deparaffinization and antigen retrieval, sections were blocked in PBS with 5% bovine serum albumin (BSA) for 1 hour and then stained overnight with the following primary antibodies: goat-anti-LepR (R&D: AF497, 10 µg/mL) and rabbit-anti-CD56 (Proteintech: 14255-1-AP, 1:2000). Next, samples were incubated with appropriate secondary antibodies, including donkey anti-goat Alexa Fluor 555 and donkey anti-rabbit Alexa Fluor 647 (all from Invitrogen, 1:400). Slides were mounted with anti-fade prolong gold (Invitrogen) and images were acquired with a Zeiss LSM780 confocal microscope.

### Cell capture and cDNA synthesis

After isolation of human CD271+ BM-MNCs, we applied the Chromium single cell gene expression platform (10x Genomics, Pleasanton, CA, USA) for scRNA-seq experiments. Cell suspensions were loaded on a Chromium Single Cell Controller (10x Genomics) to generate single-cell gel beads in emulsion (GEMs) by using Single Cell 3' Library and Gel Bead Kit V3 (10x Genomics, Cat# 1000092) and Chromium Single Cell A Chip Kit (10x Genomics, Cat#120236) according to the manufacturer's protocol. Briefly, single cells were suspended in 0.04% BSA-PBS. Cells were added to each channel, captured cells were lysed, and the released RNA were barcoded through reverse transcription in individual GEMs^27^. GEMs were reverse transcribed in a C1000 Touch Thermal Cycler (Bio Rad, Hercules, CA, USA) programmed at 53 °C for 45 min, 85 °C for 5 min, and held at 4 °C. After reverse transcription, single-cell droplets were broken, and the single-strand cDNAs were isolated and cleaned with Cleanup Mix containing DynaBeads (Thermo Fisher Scientific). cDNAs were generated and amplified, and the quality was assessed using the Agilent 4200.

### Single cell RNA-Seq library preparation

Single-cell RNA-seq libraries were prepared using Single Cell 3' Library Gel Bead Kit V3 following the manufacturer's guide (https://support.10xgenomics.com/single-cell-gene-expression/library-prep/doc/user-guide-chromium-single-cell-3-reagent-kits-user-guide-v3-chemistry). Single Cell 3' Libraries contain the P5 and P7 primers used in Illumina bridge amplification PCR. The 10x Barcode and Read 1 (primer site for sequencing read 1) were added to the molecules during the GEM-RT incubation. The P5 primer, Read 2 (primer site for sequencing read 2), Sample Index and P7 primer were added during library construction. The protocol was designed to support library construction from a wide range of cDNA amplification yields spanning from 2 ng to >2 μg without modification. Finally, sequencing was performed on an Illumina Novaseq6000 with a sequencing depth of at least 100,000 reads per cell for a 150 bp paired end (PE150) run.

### Pre-processing of scRNA-seq data

Raw FASTQ files were mapped to the Reference genome (GRCh38/hg38) using Cell Ranger 3.0 (https://support.10xgenomics.com/single-cell-gene-expression/software/pipelines/latest/what-is-cell-ranger). To create Cell Ranger-compatible reference genomes, the references were rebuilt according to instructions from 10x Genomics (https://www.10xgenomics.com), which performs alignment, filtering, barcode counting and UMI counting. Following alignment, digital gene expression (DGE) matrices were generated for each sample and for all samples. Merged 10x Genomics DGE files were generated using the aggregation function of the Cell Ranger pipeline. All cells in different batches were merged and normalized by equalizing the read depth among libraries. Only confidently mapped, non-PCR duplicates with valid barcodes and unique molecular identifiers were used to generate the gene-barcode matrix (**Figure S1A-B**). For further quality control, we excluded cells that had fewer than 150 detected genes. We then calculated the distribution of genes detected per cell and removed any cells in the top 2% quantile. We also removed cells where >20% of the transcripts were attributed to mitochondrial genes (**Figure S1C-D**). After removing disqualified cells from the dataset, the data were normalized by the total expression, multiplied by a scale factor of 10,000, and log transformed.

### Dimensionality reduction and data visualization

To visualize the data, we first calculated the ratio of binned variance to mean expression for each gene and selected the top 2,000 most variable genes. Next, we performed principal component analysis (PCA) and reduced the data to the top 20 PCs. Finally, we performed non-linear dimensionality reduction for the dataset to project the cells in 2D space based on gene expression data of the highly variable genes using t-SNE 28.

### Clustering and differential gene expression analysis

We performed a graph-based clustering of the previously identified PCs using the Louvain Method 29, and the clusters were visualized on a 2D map produced with t-SNE. For each cluster, we used the Wilcoxon rank-sum test to identify significantly differentially expressed genes (DEGs) when compared to the remaining clusters (Bonferroni correction was used to adjust for multiple hypothesis testing, adjusted *p* value < 0.05 was regarded as significant, paired tests when indicated). To visualize how well the cluster-specific DEGs (marker genes) defined each cluster, we constructed the violin plot, feature plot (tSNE plot colored by expression level of indicated genes), and heatmap (top 10 genes with highest average log-transformed fold change - logFC) using the *Seurat* R packages 30,31.

### Pathway enrichment analysis and trajectory analysis

To investigate the biological processes and signaling pathways associated with each cluster (subtype), we performed GO and KEGG enrichment analysis on the identified cluster-specific DEGs by using the *clusterProfiler* R package 32. To visualize the results, we used the *ComplexHeatmap* and *GOplot* R packages. We then applied *Monocle* for trajectory inference and pseudotime analysis 33,34. The principle of these analyses is to determine the pattern of the dynamic process experienced by the cell population and to order the cells along their developmental trajectory based on differences in the expression profiles of highly variable genes.

### Cross-species scRNA-seq data integration

Two previous independent scRNA-seq datasets of mBM-MSCs were acquired from GEO database under the accession numbers of GSE128423 and GSE108892, respectively 7,19. After acquiring the expression matrix, cells expressing LEPR were isolated as the LEPR^+^ mBM-MSC subset. We then applied canonical correlation analysis (CCA) to the top 2,000 genes with the highest dispersion shared between datasets using the Seurat alignment method to integrate scRNA-seq data of hBM-MSCs and mBM-MSCs 30,31. The CCA method identifies shared correlation structures across different datasets by finding linear combinations of the features that have large correlation. Finally, we aligned the subspaces based on the first 30 canonical correlation vectors, resulting in reduced dimensionality for further analysis 7. The batch effect was then assessed based on the correlation of average gene expression between the datasets.

## Results

### Cellular heterogeneity of the human CD271^+^ BM-MNCs

**Figure 1:**
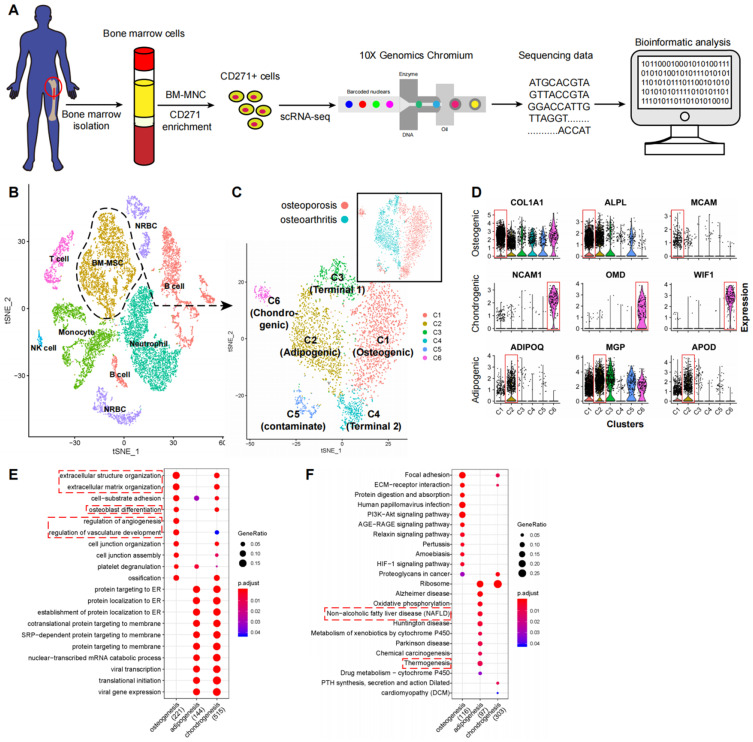
** scRNA-seq analysis of human BM-MSCs. (A)** Schematic summarizing an overview of the study. **(B-C)** t-SNE visualization of color-coded clustering of 14,494 human CD271^+^ BM-MNCs. The labeled texts indicate the individual clusters. Dashed lines in (**B**) delineate LEPR^hi^CD45^low^ BM-MSCs, which are further classified into subgroups shown in (**C**). The upper-right t-SNE plot in (**C**) shows the difference in BM-MSCs between the two subjects. **(D)** Violin plots showing relative expression levels of selected cluster-specific marker genes for osteoblast (top row), chondrocyte (middle row), and adipocyte (bottom row) precursors, respectively. **(E-F)** GO (**E**) and KEGG (**F**) enrichment analyses for osteoblast, chondrocyte, and adipocyte precursors. Dot plot shows the most significant terms. The size of dot indicates the gene ratio (enriched genes/total number of genes). The color indicates the adjusted *p* value for enrichment analysis. Dashed boxes highlight the terms related to MSC functions.

To study the transcriptomic diversity of the BM-MSCs, we applied scRNA-seq on freshly isolated CD271^+^ BM-MNCs from the femoral shaft-derived bone marrow of two human subjects (one with osteoporosis and the other with osteoarthritis) (**Figure 1A**). Cells were affinity isolated with CD271 conjugated magnetic microbeads (**see methods**), and mRNA libraries were prepared and sequenced with the 10x Genomics Chromium system. After quality filtering (**Figure S1A-C**), we obtained an expression matrix of 14,494 cells where transcripts for the average number of genes detected per cell was 1,363. There was a strong correlation between the overall gene expression profiles of the two subjects (*R* = 0.96, **Figure S1D-E**), and therefore we combined the data from the two subjects for subsequent analyses. The graph-based clustering divided the cells into 15 distinct clusters (clusters A-O), and the differentially expressed genes (DEGs) of each cluster were identified with the Wilcoxon rank-sum test (**Figure S2A-B; Table S1 Sheet 1**).

Among the cell type clusters, clusters C and D expressed high levels of BM-MSC marker genes, including LEPR (leptin receptor), NGFR (CD271), ENG (CD105), THY1 (CD90), and NT5E (CD73). Notably, LEPR had the strongest expression levels (**Figure S2C**). The remaining clusters are PTPRC (CD45) or HBA1 (hemoglobin-1) positive hematopoietic cells (**Figure S2C**). Specifically, based on the identified markers: **1)** clusters A and B are CD11b/16/66b^hi^ neutrophils; **2)** clusters F, K, and N are CD14^hi^CD16^low/hi^ monocytes; **3)** clusters E, I, L, and M are CD19^hi^ B cells; **4)** cluster H is CD3^hi^ T cells; **5)** cluster O is CD56^hi^ NK cells; and **6)** clusters G and J are HBA1^hi^ nucleated red blood cells (RBCs) (**Figure 1B; Table S1 Sheet 1**). These findings are consistent with previous reports that MSCs are the main source of LEPR expression in human bone marrow and CD271^+^ MNCs also express certain levels of CD45 (**Figure S2C**) 6,35. By comparing the gene expression pattern between LEPR^hi^CD45^low^ BM-MSCs and other CD45^hi^ hematopoietic cells, we discovered several potential surface markers for isolation of human BM-MSCs such as high expression of CD167b, CD91, CD130, CD118 and low expression or absence of CD74, CD217, CD148, CD68 (**Table S1 Sheet 2**). These results demonstrate that CD271^+^ MNCs are a heterogeneous cell population containing several cell types.

### Cellular taxonomy of BM-MSCs

To investigate the cellular heterogeneity within BM-MSCs, we extracted LEPR^+^CD45^-^ cells (clusters C and D, **Figure S2A**) from the original dataset for further analyses. The LEPR^+^CD45^-^ BM-MSCs were divided into six distinct groups by an unbiased clustering analysis (**Figure 1C and S2D**). Based on known cell markers or functional genes, the different subtypes of BM-MSCs were annotated as:** 1)** osteoblast precursor (cluster C1, expressing osteogenic markers including collagen 1 and ALPL 36,37); **2)** adipocyte precursor (cluster C2, expressing adiponectin and MGP 38,39); **3)** chondrocyte precursor (cluster C6, expressing CD56 and WIF1 14,40); and **4)** terminal-stage cells that do not express differentiation markers (clusters C3-C5) (**Figure 1D**).

We studied the expression and function of the cluster-specific DEGs in the new BM-MSCs subpopulations (**Figure 1D**, **Table S1 Sheet 3**) and found several interesting results.** 1)** Besides known markers or functional genes such as ALPL and collagen 1, some novel genes were also highly expressed in the osteoblast precursor cells. For instance, MCAM (CD146) was differentially expressed in osteoblast precursors when compared with other cell subtypes. CD146 was recently reported as one of the markers for human osteoblast precursors 15. **2)** Along with ADPQ (adiponectin) and MGP, APOD (apolipoprotein D) was also highly expressed in the adipocyte precursors. **3)** Osteomodulin (OMD) was highly expressed in the chondrocyte precursors. Previous reports have shown that OMD induces endochondral ossification through PI3K signaling, and regulates the extracellular matrix during bone formation by reorganizing collagen fibrils and increasing aggrecan expression in chondrocytes 41-43. Taken together, the findings suggest that OMD may potentially regulate chondrogenic differentiation.

To study the shared and distinct biological processes between different cell type clusters, we performed GO and KEGG functional term enrichment analysis of DEGs in osteoblast, chondrocyte, and adipocyte precursors (**Table S2**). Several enrichment terms for bone development were detected in the osteoblastic and chondrocyte precursors including “ossification”, “osteoblast differentiation”, etc. The adipocyte precursors were enriched for terms such as “non-alcoholic fatty liver disease” and “thermogenesis” (**Figure 1E-F**) 44,45. These results demonstrate that human BM-MSCs consist of a heterogeneous cell population with several different subtypes, which are characterized by distinct biological processes.

In contrast, the remaining subgroups (clusters C3-C5) of the BM-MSCs did not express any differentiation markers, and the GO enrichment analyses did not detect any significant terms related to differentiation processes. Members of ribosomal protein (RP) gene family, which encodes ribonucleoprotein, were highly expressed in clusters C3 and C4 (**Table S1 Sheet 3**). Previous evidence suggests that the expression of ribonucleoprotein is required for maintenance of self-renewal potency of stem cells 46. These clusters were enriched for GO terms related to ribonucleoprotein, RNA degeneration, and cell apoptosis (**Figure S3A**). These results partially support the claim that these clusters contain cells at terminal stage which lack the capacity for cellular differentiation. We noted that although cluster C5 had high expression levels of LEPR, a small fraction of the cells in this group also expressed low levels of CD45 and were enriched for immune cell related terms such as “neutrophil cell activation” and “leukocyte migration” (**Figure S2E and S3A**). This suggests that CD45^+^ immune cells may have contaminated this cluster, and therefore we excluded this cluster (C5) from further analysis.

### Dynamic gene expression patterns at different developmental stages of BM-MSCs

**Figure 2:**
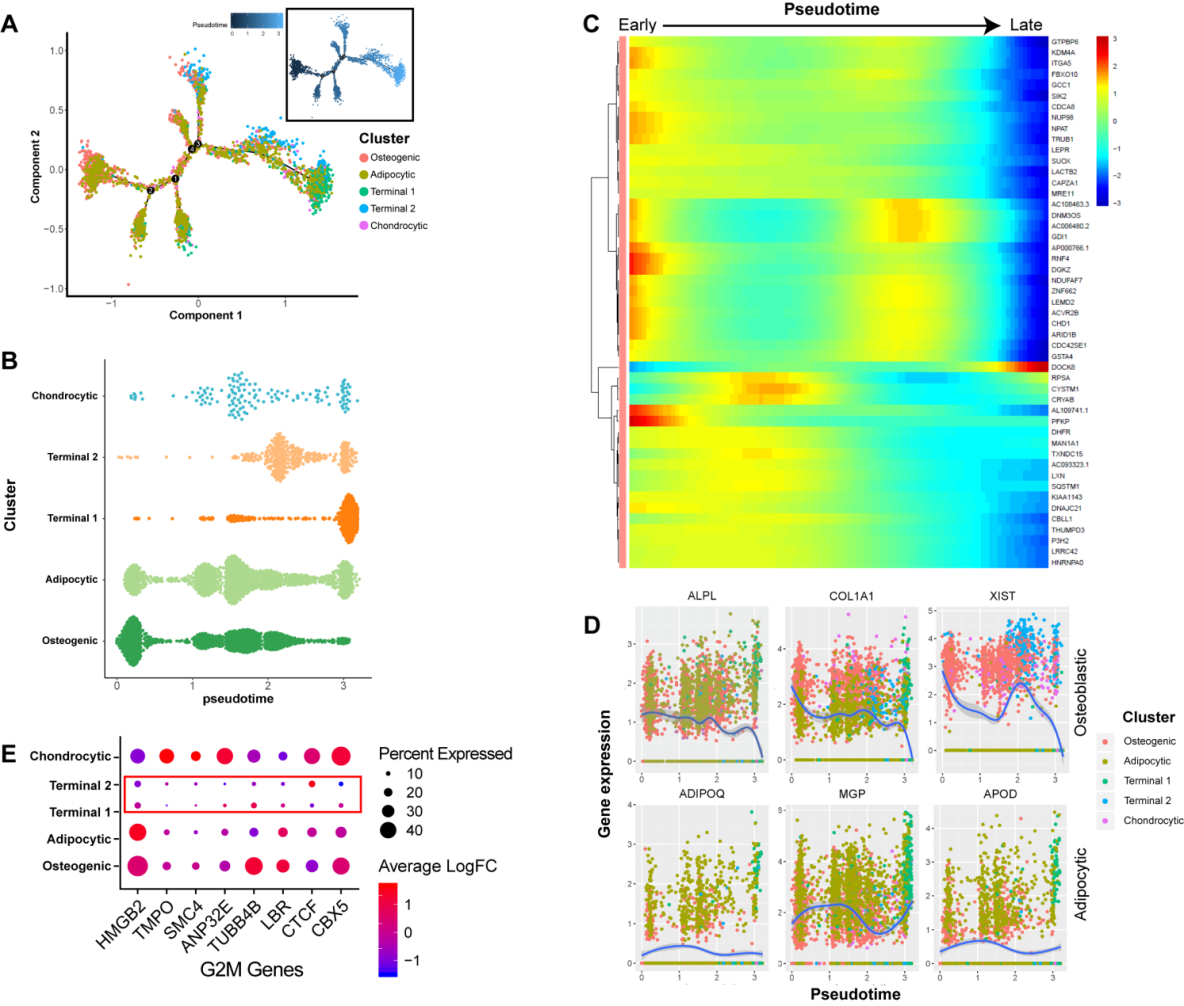
** Dynamic gene expression patterns of human BM-MSCs. (A)** Reconstructed principal component graph of cell differentiation trajectory of BM-MSCs, colored by subpopulation identities. The upper-right trajectory plot in the square indicates the direction of pseudotime. **(B)** Distribution of each cell subpopulation along the pseudotime. **(C)** Continuum of dynamic gene expression in pseudotime of BM-MSCs. Heatmap shows top 50 genes with most significant expression changes. Pixel color indicates the expression level (logFC).** (D)** Expression level of osteogenic (top) and adipogenic (bottom) genes with respect to pseudotime coordinates. Blue lines depict the LOESS regression fit of the normalized expression values. **(E)** Expression pattern of G2M genes of BM-MSCs.

In order to better understand the differentiation relationships between BM-MSCs subtypes, we reconstructed the developmental trajectory by inferring the dynamic gene expression patterns at different developmental stages. The estimated developmental trajectory showed multiple branches, representing the multi-lineage differentiation potential of BM-MSCs (**Figure 2A**). By comparing the distribution of the cell population along the pseudotime, we found that osteoblast precursors (cluster C1) were more enriched in the early stage of pseudotime compared with the other clusters, while adipocyte and chondrocyte cells were evenly distributed along the pseudotime (**Figure 2B**). Pseudotime ordering of cell type clusters revealed a continuum of gene expression between the early and late stages of BM-MSC differentiation (**Figure 2C**). When the dynamic gene expression patterns between osteoblast and adipocyte markers were compared, the osteoblast markers decreased over pseudotime, while the adipocyte markers remained unchanged or increased (**Figure 2D**). These findings suggest that osteoblast precursors are only differentiated at the early stage of BM-MSC development, while adipogenesis is continuous across different stages. We also noticed that clusters C3 and C4 were mostly represented at the later stage of the pseudotime (**Figure 2B**). By analyzing the gene expression pattern, we found that G2M genes 47 were expressed at lower levels in these two clusters (**Figure 2E**).

### Osteoblast precursors induce angiogenesis during coupling with osteogenesis

**Figure 3:**
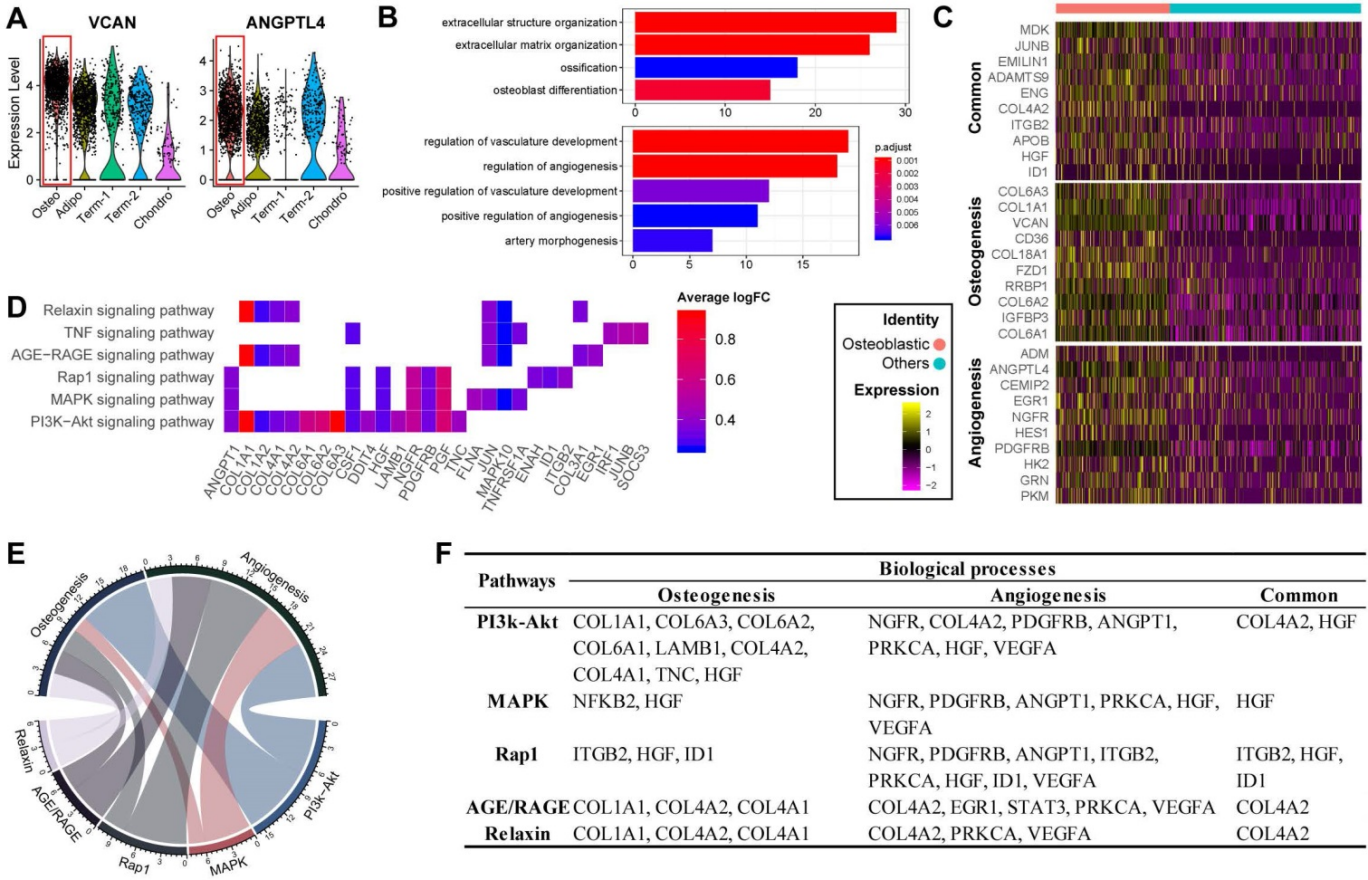
** Functional analysis for ALPL^hi^ osteoblast precursor. (A)** Violin plots showing relative expression levels of VCAN and ANGPTL4. Osteoblastic cluster was highlighted by the red box. **(B)** Enriched GO terms associated with osteogenesis (top) and angiogenesis (bottom) in osteoblast precursors. Bar chart shows the number of genes enriched in each term. Color indicates the adjust* p* values. **(C)** Differential expression of osteogenesis- and (or) angiogenesis-related genes (rows) in osteoblast precursors compared to the other cells. Heatmap shows top 10 most significant DEGs in each category, where color indicates the relative expression levels between osteoblast precursors and other cells (z-score). **(D)** Gene expression pattern in enriched pathways. Squares show enriched DEGs in the corresponding terms (rows). Color indicates the expression value of the DEGs (average logFC). **(E-F)** Table of genes in biological processes and pathways. **(E)** Numbers outside the circles indicate the number of genes in that term. Width of curves connecting different terms is proportional to the number of shared genes.** (F)** Table of the specific genes enriched in each biological process and pathway.

Previous studies have reported that osteoblasts may regulate angiogenesis 48,49, but this phenomenon has not yet been explored at the single-cell level. Interestingly, transcripts for some secreted factors associated with the vascular system (e.g., VCAN and ANGPTL4 50,51) were highly expressed in the osteoblast precursors, (**Figure 3A**). This result suggests that osteoblast precursors may induce angiogenesis concurrently with osteogenesis. In supporting this, the cluster marker genes of osteoblast precursors were enriched for not only osteogenesis related GO terms, but also for functional processes related to angiogenesis such as “regulation of vasculature development” and “positive regulation of angiogenesis” (**Figure 1E and 3B**). We further investigated the genes enriched for these biological processes and identified 32 genes regulating osteogenesis (e.g., COL1A1/A3, COL6A1/A3, VCAN, IGFBP3, etc.), 16 genes for angiogenesis (e.g., ADM, EGR1, NGFR, etc.), and 11 shared genes including MDK, JUNB, ENG, IGTB2, APOB, etc. (**Figure 3C; Table S3 Sheet 1**). Among these genes, some have a much higher expression level in the osteoblast precursors compared with other cells.

Notably, we found that MDK, CD105, and ADAMTS9 were highly expressed and frequently enriched in multiple functional terms related to osteogenesis and angiogenesis (**Figure S3B**). It has been shown that MDK is positively associated with angiogenesis while inversely associated with osteogenesis 52,53, potentially via MAPK and PI3K signaling 54. High expression of CD105 has been shown to disrupt angiogenesis in tumor tissue, and CD105^-^ BM-MSCs are more prone to differentiate into adipocytes and osteocytes 11,55. ADAMTS9 is expressed during ossification and also may regulate angiogenic signaling induced by VEGF 56,57. Our results together with the previous evidence suggest that the co-regulation of osteogenesis and angiogenesis by osteoblast precursors is a complex network involving multiple genes whose regulatory effects are sometimes in opposite directions.

The KEGG pathway analysis revealed that the osteogenesis and angiogenesis genes were enriched in the PI3K-Akt, MAPK, Rap1, AGE-RAGE, Relaxin, and TNF signaling pathways, in which PI3K-Akt signaling had the most genes enriched (**Figure 3D**). The genes COL1A1, PGF, and JUN were highly expressed and were also enriched in multiple pathways, indicating that these genes may be essential in the various cell signaling networks. We also found that PI3K-Akt signaling and osteogenesis share a large proportion of common genes, suggesting that this pathway may have a significant role in regulating osteogenesis of BM-MSCs (**Figure 3E**). On the other hand, the MAPK, PI3K-Akt, and Rap1 signaling pathways share comparable proportions of genes with angiogenesis (**Figure 3E**). Furthermore, COL4A2, HGF, IGBT1, and ID1 are essential factors connecting the genetic network between the different pathways and biological processes (**Figure 3F**). These results suggest that osteogenesis and angiogenesis in osteoblast precursors are likely mediated by multiple genes and pathways, and particularly through PI3K-Akt signaling pathways.

### Myogenesis potential of CD56^+^ chondrocyte precursors

**Figure 4:**
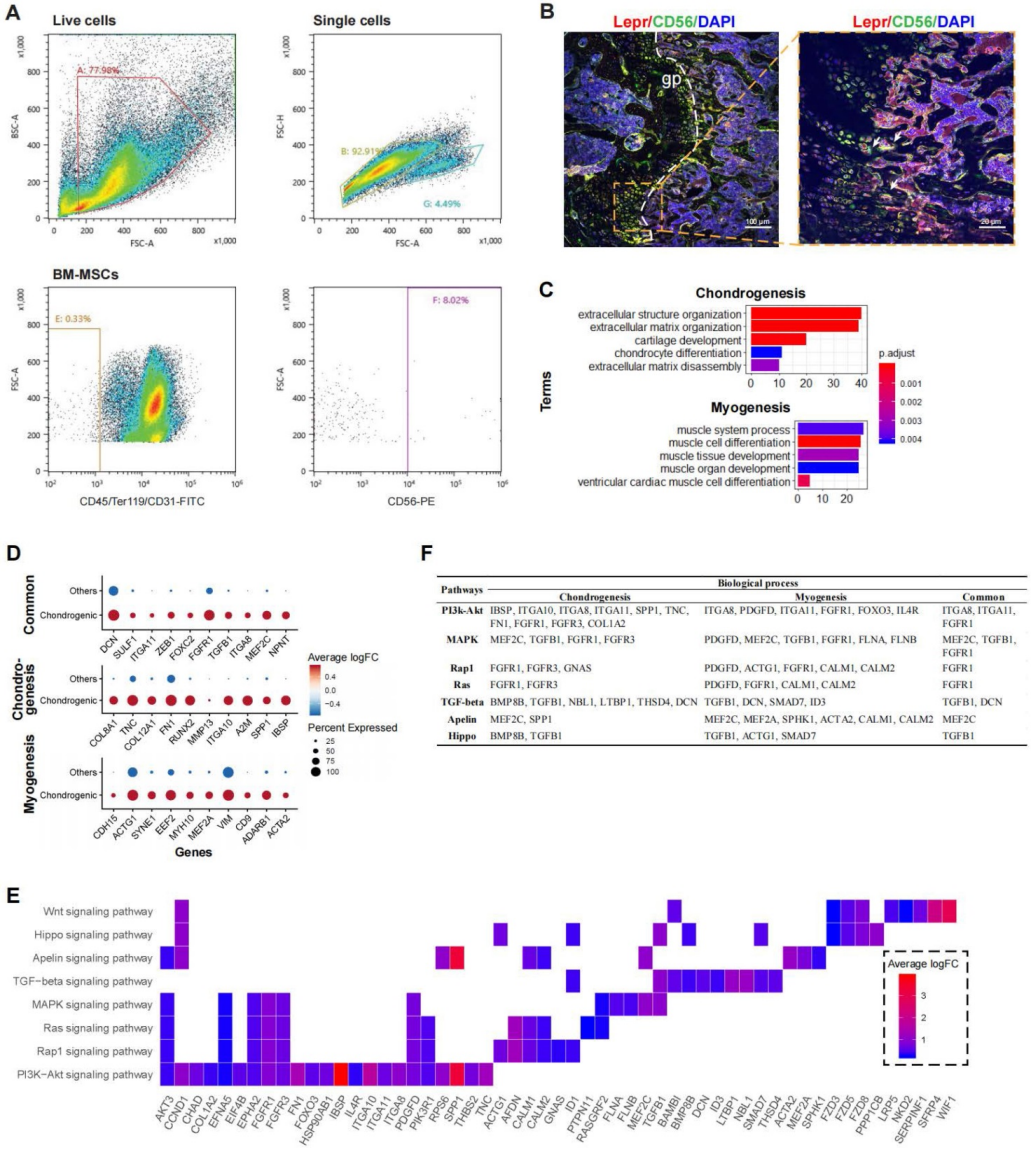
** Functional analysis for CD56hi chondrocyte precursor. (A)** Flow cytometry dot plot showing proportion of CD56^+^ BM-MSC fraction in mouse. **(B)** Representative confocal immunofluorescent imaging showing distribution of Lepr^+^CD56^+^ BM-MSCs in murine femur at low (left) and high (right) magnification. Arrows marked Lepr^+^CD56^+^ cells **(C)** Enriched GO terms associated with chondrogenesis (top) and myogenesis (bottom) in chondrocyte precursor cells. Bar chart shows the number of enriched genes in each term. Color indicates the adjust *p* values. **(D)** Differential expression of chondrogenesis- and (or) myogenesis-related genes in chondrocyte precursors compared to the other cells. Dot plot shows top 10 most-significant DEGs in each category (Middle: Chondrogenesis; Bottom: Myogenesis; Top: Common for both), where dot color indicates the relative expression levels between chondrocyte precursors and other cells (z-score) and the dot size shows the proportion of cells expressing the indicated genes. **(E)** Gene expression pattern in enriched pathways. Squares show enriched DEGs in the corresponding terms (rows). Color indicates the expression value of the DEGs (average logFC). **(F)** Table of genes in biological processes and pathways.

To partially confirm the existence of the CD56^+^ BM-MSCs, we performed flow cytometry analysis on murine bone marrow derived cells. The result confirmed that CD56^+^ fraction makes up about 8% of the total BM-MSCs (**Figure 4A**). Confocal immunofluorescence imaging of murine femur further demonstrated that CD56^+^ BM-MSCs (Lepr^+^CD56^+^ cells) were mainly located at the growth plate (**Figure 4B and S3E**). The DEGs in CD56^+^ chondrocyte precursors were enriched in GO terms related to both chondrogenesis (e.g., “cartilage development”, “chondrocyte differentiation”) and myogenesis (e.g., “muscle cell differentiation”, “myoblast differentiation”) (**Figure 4C**). There were 46 DEGs enriched in terms related to chondrogenesis (e.g., IBSP, SPP1, A2M, IGTA10, etc.), 42 for myogenesis (e.g., ACTA2, ADARB1, CD9, VIM, etc.), and 13 shared genes for both processes (e.g., NPNT, MEF2C, ITGA8, TGFB1, etc.) (**Figure 4D and S3C**).

Based on the KEGG pathway analysis, we determined that DEGs in the chondrocyte precursors were enriched in the PI3K-Akt, MAPK, Ras, Rap1, TGF-beta, Apelin, and Hippo signaling pathways (**Figure 4E**). TGF-beta signaling shared the largest number of genes with chondrogenesis, while the genes enriched in Apelin and Ras/Rap1 signaling overlapped mostly with myogenesis (**Figure S3D**). By investigating the overlapping genes between biological processes and signaling pathways, we found that FGFR1 and TGFB1 may be crucial genes connecting multiple pathways to both chondrogenesis and myogenesis (**Figure 4F**). Thus, the CD56^+^ chondrocyte precursor of the BM-MSC subpopulation is capable of both chondrogenesis and myogenesis, and these processes may be regulated by the TGF-beta, Apelin, and Ras/Rap1 signaling pathways.

### Transcriptional difference between human and mice BM-MSCs at single-cell level

**Figure 5:**
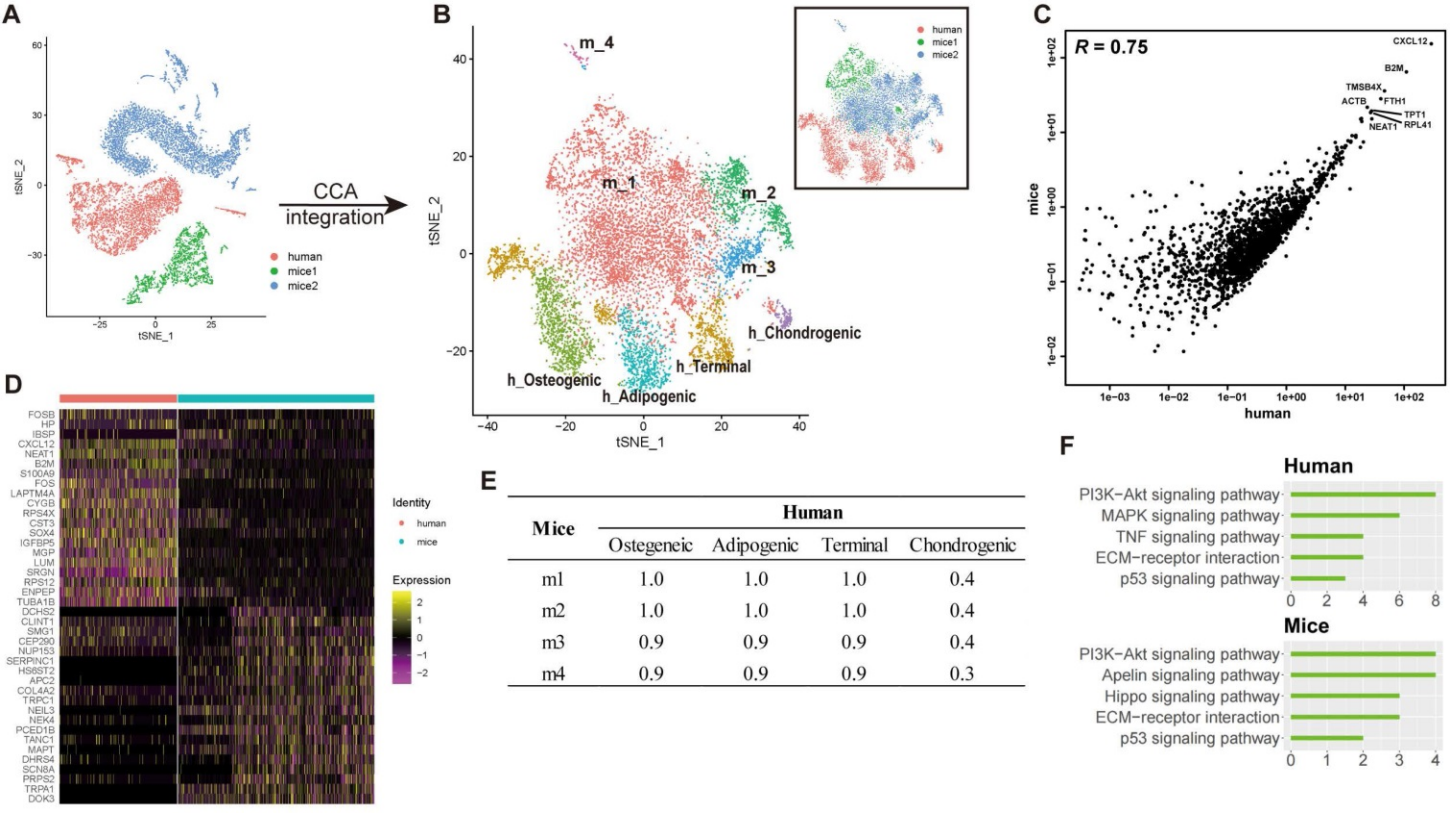
** Integrated cross-species analysis between human and mouse BM-MSCs.** (**A-B**) t-SNE visualization of human and mouse BM-MNCs before (**A**) and after (**B**) CCA integration. The labeled texts indicate the datasets or subpopulations identified by clustering analysis. Human (h): data from this study; mice1 (m): data from Tikhonova et al. 7; mice2 (m): data from Baryawno et al. 19. (**C**) Correlations of gene expression among human and mouse BM-MSCs after CCA integration. Each dot represents an individual gene. Texts indicate highly expressed genes shared between the two species. The average gene expression level is plotted for each subject. Correlations were measured by Pearson correlation coefficients (*R*, p < 0.01). (**D**) Gene signature of human and mouse BM-MSCs, based on the relative gene expression level of top 20 most-significant DEGs for each species (z-score). (**E**) Correlations of gene expression between different subsets of human and mouse BM-MSCs identified by clustering analysis (Osteogenic, chondrogenic, adipogenic and terminal in human; m1-m4 in mice). Values in the table represent the Pearson correlation coefficients (*R*, p < 0.01). (**F**) Enriched signal pathways (KEGG terms) of human (top) and mice (bottom) BM-MSCs. Bar chart shows the number of enriched genes in each term.

To investigate the difference of transcriptional profiles between BM-MSCs acquired from humans and mice (hBM-MSCs, mBM-MSCs, respectively), we integrated our single-cell human transcriptome data with two previous scRNA-seq studies of bone marrow components in mice 7,19. Potential batch effects among different studies were reduced by canonical correlation analysis (CCA) (**see methods**) 58,59, and the transcriptomic profiles from different datasets had high correlation (**Figures 5A-C and S4A**), suggesting that after the CCA integration, the batch effects between different studies were relatively small and were, therefore, unlikely to introduce notable bias into the downstream analysis.

To test whether heterogeneity exists between human and mice BM-MSCs, the integrated cross-species data were analyzed by an unbiased clustering. hBM-MSCs and mBM-MSCs were separated into different clusters (osteogenic, chondrogenic, adipogenic, and terminal in human; m1-m4 in mice) (**Figure 5B**). We also observed significant differences in the gene expression pattern between human and mice BM-MSCs at single-cell level (**Figure 5D, Table S1 Sheet 4**). The clustering and gene expression results suggested that even though the overall data had a large correlation based on average gene expression, there were still systematic differences between hBM-MSCs and mBM-MSCs transcriptomes at the single-cell level. There was a strong correlation between the average gene expression of subtypes in hBM-MSCs and mBM-MSCs except for human chondrogenic BM-MSCs (**Figure 5E**). This observation suggests that the overall gene expression pattern and differentiation trajectory of hBM-MSC derived chondrocyte precursors is less similar with those in the mBM-MSCs, when compared to other hBM-MSC subpopulations.

Human and mice BM-MSCs often present different cell surface markers 3. Consistent with this result, by comparing the DEGs between hBM-MSCs and mBM-MSCs, we revealed several CD markers with distinct expression patterns between human and mice BM-MSCs. For instance, CD317, CD36, and CD63 were highly expressed in hBM-MSCs, but not in mBM-MSCs; and *vice versa* for CD148, CD108, and CD20 (**Figure S4B**).

## Discussion

While a growing body of evidence indicates that BM-MSCs have a central role in bone health, the underlying subtypes of BM-MSCs, especially *in vivo* in humans, remains largely unknown due to its heterogeneous characteristics. In the present study, we applied scRNA-seq analysis on freshly isolated human BM-MSCs and their niche hematopoietic cells. The use of freshly isolated human cells is a major advantage of this study, since any form of extra *in vitro* operations (e.g., freezing, culturing) could potentially alter the true transcription pattern 22 and thus lead to biased cell clustering/identification. In addition, our results along with previous evidence have highlighted that transcription profiles vary to a large degree between humans and mice 23.

Several studies have applied scRNA-seq on bone marrow stroma components or MSCs derived from various origins (e.g., bone marrow, adipocytes, umbilical cord). For instance, Tikhonova et al. 7 and Baryawno et al. 19 independently performed scRNA-seq on bone marrow stroma components (including BM-MSCs, vasculature, osteoblastic cells, etc.). Similar to their results, we also identified subtypes corresponding to multiple trajectories in BM-MSCs. Chan et al. 15,16, on the other hand, identified skeletal stem cells in humans and mice. They also demonstrated a Lin^-^PDPN^-^CD146^+^ osteogenic subset that only gives rise to osteoblasts/osteocytes 15. Similarly, we found that CD146 was differentially expressed in the osteogenic subset of BM-MSCs. Some studies also performed scRNA-seq on cultured human MSCs derived from various origins 20,21,62, but none of these studies focused on subtype identification. Compared with those studies that focused on mouse cells or *in vitro* cultured human cells, our results thus greatly expand the understanding of *in vivo* human BM-MSCs by presenting unbiased transcriptional profiles of distinct subpopulations including osteoblast, chondrocyte and adipocyte precursors as well as other components of the human BM-MSC cell population *in vivo*.

Although the use of freshly isolated cells for scRNA-seq may preserve to the largest extent the accuracy of the transcriptomic profile, this approach also limits the total number of collected cells. Therefore, we used a single marker - CD271 - for positive sorting, instead of combining with CD45-negative selection, which would generate even less yield. Based on the scRNA-seq gene expression profiles, we demonstrated that the CD271^+^ BM-MNCs represent a heterogeneous cell population, which may be subdivided into BM-MSCs along with various hematopoietic cells that contribute to the formation of niche components. Our finding suggests that the BM-MSC isolation protocol based solely on positive selection is not ideal as the isolated cells consist of various cell types. Instead, positive selection combined with negative selection using CD45 or lineage markers (LIN) should be considered if the major purpose is to isolate BM-MSCs with a higher purity 5,63.

Interestingly, though we used CD271 as the cell surface marker for BM-MSC positive selection, the gene expression of CD271 was lower than expected (**Figure S2C**), suggesting that the protein expression may not be associated with the expression of the corresponding gene. Previous single-cell studies also showed similar results. For instance, Qin et al.'s results 64,65 showed that even after positive selection of Col2^+^ cells by FACS sorting, the single-cell gene expression of Col2 (Col2a1) is lower than expected. It is well known that the abundance of expressed proteins cannot always be inferred directly from mRNA readout alone 66. New single-cell techniques have emerged which can simultaneously evaluate gene expression at both transcript and protein level, which may provide a more accurate characterization of cellular identity, states, and function 67.

Since BM-MSCs are heterogeneous for several existing cell markers 7,9, it is necessary to search for novel BM-MSC-specific cell markers (specifically and uniformly expressed in the major BM-MSC populations). By comparing the expression pattern between BM-MSCs and other niche hematopoietic cells, we confirmed the expression of classic cell markers including CD271, LEPR, CD105, and CD90 at the single-cell level. Notably, we found that LEPR had the highest expression level and was specific to the BM-MSC population, which is consistent with the results from mouse models 35. We also detected some additional specifically expressed CD markers (e.g., CD167b, CD91, CD130, CD118) in BM-MSCs, which may potentially serve as novel surface markers for BM-MSC enrichment/purification.

A systematic analysis of the BM-MSC transcriptional profiles revealed distinct subpopulations corresponding to osteogenic, chondrogenic, and adipogenic differentiation, as well as terminal-stage cells in the quiescent state. Further examination into the relationships between the highly expressed genes, biological processes, and signaling pathways in each subpopulation suggests that osteoblast precursors may have the capacity to induce vasculature development, and the chondrocyte precursors may have myogenic potential. Normally, the coupling of osteogenesis and angiogenesis is in the same regulation direction, i.e., vascular development will promote bone formation and *vice versa* 68. However, several recent studies have already shown that in some cases the regulatory effect of these two biological processes could be opposite. For instance, even though VEGF stimulates vascularization, high amounts of VEGF could impair bone formation 69. Similar patterns were found in BM-MSCs in this study where osteoblast precursors express CD105 and MDK, whose regulatory effect on osteogenesis and angiogenesis may be opposite, suggesting that the coupling of osteogenesis and angiogenesis is a complex regulatory network where both positive and negative feedback may be included. It has been proposed that bone and muscle act as secretory endocrine organs affecting the function of one another through various pleiotropic genes and signaling pathways including (e.g., FGF-2, FGF-23, TGF-β, Wnt-3a) 70-72. Our results demonstrated similar findings. For instance, we found that Wnt and TGF pathways may have important roles in chondrogenic-myogenic crosstalk in BM-MSCs. We also found that FGF receptor may contribute to the crosstalk through various pathways such as MAPK and Ras.

Some interesting results were discovered when we analyzed the subtypes of BM-MSCs in-depth. We found that APOD was highly expressed in the adipocytic subtypes. Although APOD has not previously been linked to adipogenesis, other apolipoproteins, such as APOA and APOE 73,74 are known to modulate adipocyte metabolism. Therefore, it is conceivable that APOD may also regulate adipogenesis. We found that G2M genes were less expressed in the terminal stage BM-MSCs. Although this result somewhat suggested that the terminal cells might be quiescent stem cells, the stem cell markers were not differentially expressed in terminal cells. Therefore, the identity of terminal cells remains elusive, and worth further investigation. The scRNA-seq profiles of the BM-MSCs also revealed a continuum of dynamic gene expression pattern, indicating that osteogenesis occurs only at the early stages of BM-MSC development while the adipogenic and quiescent cells take a dominant place in the terminal stages (**Figure 2B**). These findings suggest that aging of BM-MSCs represents an important factor in the balance between the osteogenic and adipogenic differentiation.

Although emerging studies have explored the single-cell transcriptome of both human and mouse BM-MSCs, few have considered the cross-species difference of transcriptome between h/m-BM-MSCs. Several studies have described such differences in other tissues at single-cell level 75,76. By integrating our hBM-MSCs data with previous scRNA-seq data of mBM-MSCs, we were able to systematically analyze the shared and specific features of h/m-BM-MSCs. The findings suggest that some features are conserved across species, such as the high expression of Cxcl12, while other features such as the surface markers and genes regulating osteo-/adipo-genesis may be different. Understanding the systematic differences between h/m-BM-MSCs is essential, especially when attempting to adapt the conclusions from mouse models to humans or *vise versa*.

We also note some limitations of our study design. Firstly, these findings are based on bioinformatic analysis of single-cell transcriptome, and without further molecular validations, some of these results are suggestive rather than conclusive, such as proposed cell markers or differentiation potentials. Other limitations of this study include batch effect and sample size. While the overall data did not show a significant batch effect, the transcription pattern of the BM-MSCs varied between the two human subjects (**Figure S2D**). We hypothesize that this may be explained by the gender and age differences. However, with limited sample size, it is difficult to deduce whether and/or how such differences are related to the disease status (e.g., osteoporosis vs. osteoarthritis) or other factors (e.g., age, gender, lifestyle, medical/medication history). In future studies, more subjects should be included to overcome potential batch effects and to explore how different health states and other factors affect the bone marrow microenvironment.

Despite providing a detailed characterization of human BM-MSCs at single-cell resolution, the full trajectory of the osteoblastic lineage cells, as well as their balance and interaction with the osteoclastic lineage remain elusive. In our future studies, by combining scRNA-seq with scATAC-seq - a powerful tool to evaluate chromatin accessibility at the single-cell level, we will aim to unveil the complexity of osteoblastic-osteoclastic lineage interactions and gene expression regulations within/between the two lineages. In the meantime, deconvoluting the heterogeneity of BM-MSCs *in vivo* in humans represents an important and necessary advancement towards improving our understanding of bone physiological processes.

## Supplementary Material

Supplementary figures and table legends.

Supplementary table 1.

Supplementary table 2.

Supplementary table 3.

## References

[1] Boyle WJ, Simonet WS, Lacey DL (2003). Osteoclast differentiation and activation. Nature.

[2] Demontiero O, Vidal C, Duque G (2012). Aging and bone loss: New insights for the clinician. Vol. 4, Therapeutic Advances in Musculoskeletal Disease.

[3] Ambrosi TH, Longaker MT, Chan CKF (2019). A Revised Perspective of Skeletal Stem Cell Biology. Vol. 7, Frontiers in Cell and Developmental Biology. Frontiers Media S.A.

[4] Pontikoglou C, Deschaseaux F, Sensebé L, Papadaki HA (2011). Bone Marrow Mesenchymal Stem Cells: Biological Properties and Their Role in Hematopoiesis and Hematopoietic Stem Cell Transplantation. Stem Cell Rev Reports.

[5] Quirici N, Soligo D, Bossolasco P, Servida F, Lumini C, Deliliers GL (2002). Isolation of bone marrow mesenchymal stem cells by anti-nerve growth factor receptor antibodies. Exp Hematol.

[6] Poloni A, Maurizi G, Rosini V (2009). Selection of CD271+ cells and human AB serum allows a large expansion of mesenchymal stromal cells from human bone marrow. Cytotherapy.

[7] Tikhonova AN, Dolgalev I, Hu H (2019). The bone marrow microenvironment at single-cell resolution. Nature.

[8] Yang M, Arai A, Udagawa N (2017). Osteogenic Factor Runx2 Marks a Subset of Leptin Receptor-Positive Cells that Sit Atop the Bone Marrow Stromal Cell Hierarchy. Sci Rep.

[9] Akiyama K, You Y-O, Yamaza T (2012). Characterization of bone marrow derived mesenchymal stem cells in suspension. Stem Cell Res Ther.

[10] Yamamoto N, Akamatsu H, Hasegawa S (2007). Isolation of multipotent stem cells from mouse adipose tissue. J Dermatol Sci.

[11] Anderson P, Carrillo-Gálvez AB, García-Pérez A, Cobo M, Martín F (2013). CD105 (Endoglin)-Negative Murine Mesenchymal Stromal Cells Define a New Multipotent Subpopulation with Distinct Differentiation and Immunomodulatory Capacities. Menendez P, Ed. PLoS One.

[12] Dominici M, Le Blanc K, Mueller I (2006). Minimal criteria for defining multipotent mesenchymal stromal cells. The International Society for Cellular Therapy position statement. Cytotherapy.

[13] Madsen SD, Russell KC, Tucker HA (2017). Decoy TRAIL receptor CD264: a cell surface marker of cellular aging for human bone marrow-derived mesenchymal stem cells. Stem Cell Res Ther.

[14] Battula VL, Treml S, Bareiss PM (2009). Isolation of functionally distinct mesenchymal stem cell subsets using antibodies against CD56, CD271, and mesenchymal stem cell antigen-1. Haematologica.

[15] Chan CKF, Gulati GS, Sinha R (2018). Identification of the Human Skeletal Stem Cell. Cell. 2018/09/23.

[16] Chan CKF, Seo EY, Chen JY (2015). Identification and specification of the mouse skeletal stem cell. Cell.

[17] Hedlund E, Deng Q (2018). Single-cell RNA sequencing: Technical advancements and biological applications. Mol Aspects Med.

[18] Hwang B, Lee JH, Bang D (2018). Single-cell RNA sequencing technologies and bioinformatics pipelines. Exp Mol Med.

[19] Baryawno N, Przybylski D, Kowalczyk MS (2019). A Cellular Taxonomy of the Bone Marrow Stroma in Homeostasis and Leukemia. Cell.

[20] Freeman BT, Jung JP, Ogle BM (2015). Single-cell RNA-Seq of bone marrow-derived mesenchymal stem cells reveals unique profiles of lineage priming. PLoS One.

[21] Barrett AN, Fong CY, Subramanian A (2019). Human Wharton's Jelly Mesenchymal Stem Cells Show Unique Gene Expression Compared with Bone Marrow Mesenchymal Stem Cells Using Single-Cell RNA-Sequencing. Stem Cells Dev.

[22] Neumann E, Riepl B, Knedla A (2010). Cell culture and passaging alters gene expression pattern and proliferation rate in rheumatoid arthritis synovial fibroblasts. Arthritis Res Ther.

[23] Lin S, Lin Y, Nery JR (2014). Comparison of the transcriptional landscapes between human and mouse tissues. Proc Natl Acad Sci U S A.

[24] Xie H, Sun M, Liao XB (2011). Estrogen receptor α36 mediates a bone-sparing effect of 17β-estrodiol in postmenopausal women. J Bone Miner Res.

[25] Kanis JA, Melton LJ, Christiansen C, Johnston CC, Khaltaev N (1994). The diagnosis of osteoporosis. J Bone Miner Res.

[26] Wu XP, Liao EY, Zhang H, Shan PF, Cao XZ, Liu SP (2004). Establishment of BMD reference plots and determination of peak BMD at multiple skeletal regions in mainland Chinese women and the diagnosis of osteoporosis. Osteoporos Int.

[27] Zheng GXY, Terry JM, Belgrader P (2017). Massively parallel digital transcriptional profiling of single cells. Nat Commun.

[28] García-Alonso CR, Pérez-Naranjo LM, Fernández-Caballero JC (2014). Multiobjective evolutionary algorithms to identify highly autocorrelated areas: The case of spatial distribution in financially compromised farms. Ann Oper Res.

[29] Blondel VD, Guillaume JL, Lambiotte R, Lefebvre E (2008). Fast unfolding of communities in large networks. J Stat Mech Theory Exp. 2008.

[30] Satija R, Farrell JA, Gennert D, Schier AF, Regev A (2015). Spatial reconstruction of single-cell gene expression data. Nat Biotechnol.

[31] Stuart T, Butler A, Hoffman P (2019). Comprehensive Integration of Single-Cell Data. Cell.

[32] Yu G, Wang LG, Han Y, He QY (2012). ClusterProfiler: An R package for comparing biological themes among gene clusters. Omi A J Integr Biol.

[33] Ji Z, Ji H (2016). TSCAN: Pseudo-time reconstruction and evaluation in single-cell RNA-seq analysis. Nucleic Acids Res.

[34] Trapnell C, Cacchiarelli D, Grimsby J (2014). The dynamics and regulators of cell fate decisions are revealed by pseudotemporal ordering of single cells. Nat Biotechnol.

[35] Zhou BO, Yue R, Murphy MM, Peyer JG, Morrison SJ (2014). Leptin-receptor-expressing mesenchymal stromal cells represent the main source of bone formed by adult bone marrow. Cell Stem Cell.

[36] Wennberg C, Hessle L, Lundberg P (2000). Functional characterization of osteoblasts and osteoclasts from alkaline phosphatase knockout mice. J Bone Miner Res.

[37] Shi S, Kirk M, Kahn AJ (2009). The role of type I collagen in the regulation of the osteoblast phenotype. J Bone Miner Res.

[38] Fu Y, Luo N, Klein RL, Timothy Garvey W (2005). Adiponectin promotes adipocyte differentiation, insulin sensitivity, and lipid accumulation. J Lipid Res.

[39] Mutch DM, Rouault C, Keophiphath M, Lacasa D, Clément K (2009). Using gene expression to predict the secretome of differentiating human preadipocytes. Int J Obes.

[40] Surmann-Schmitt C, Widmann N, Dietz U (2009). Wif-1 is expressed at cartilage-mesenchyme interfaces and impedes Wnt3a-mediated inhibition of chondrogenesis. J Cell Sci.

[41] Juchtmans N, Dhollander AAM, Coudenys J (2015). Brief report: Distinct dysregulation of the small leucine-rich repeat protein family in osteoarthritic acetabular labrum compared to articular cartilage. Arthritis Rheumatol.

[42] Guntur AR, Rosen CJ, Naski MC (2012). N-cadherin adherens junctions mediate osteogenesis through PI3K signaling. Bone.

[43] Tashima T, Nagatoishi S, Sagara H, Ohnuma SI, Tsumoto K (2015). Osteomodulin regulates diameter and alters shape of collagen fibrils. Biochem Biophys Res Commun.

[44] Song NJ, Chang SH, Li DY, Villanueva CJ, Park KW (2017). Induction of thermogenic adipocytes: Molecular targets and thermogenic small molecules. Vol. 49, Experimental and Molecular Medicine. Nature Publishing Group.

[45] Parker R (2018). The role of adipose tissue in fatty liver diseases. Vol. 2, Liver Research. KeAi Communications Co.

[46] Chen Q, Jin M, Zhu J, Xiao Q, Zhang L (2013). Functions of Heterogeneous Nuclear Ribonucleoproteins in Stem Cell Potency and Differentiation. Biomed Res Int.

[47] Nestorowa S, Hamey FK, Pijuan Sala B (2016). A single-cell resolution map of mouse hematopoietic stem and progenitor cell differentiation. Blood.

[48] Li J, Zhang Y, Zhao Q, Wang J, He X (2015). MicroRNA-10a influences osteoblast differentiation and angiogenesis by regulating β-catenin expression. Cell Physiol Biochem.

[49] Liu C, Cui X, Ackermann TM, Flamini V, Chen W, Castillo AB (2016). Osteoblast-derived paracrine factors regulate angiogenesis in response to mechanical stimulation. Integr Biol (United Kingdom).

[50] Evanko SP, Chan CK, Johnson PY, Frevert CW, Wight TN (2018). The biochemistry and immunohistochemistry of versican. In: Methods in Cell Biology. Academic Press Inc.

[51] Huang S, Wang M, Rehman MU (2018). Role of Angiopoietin-like 4 on Bone Vascularization in Chickens Exposed to High-altitude Hypoxia. J Comp Pathol.

[52] Lautz T, Lasch M, Borgolte J (2018). Midkine Controls Arteriogenesis by Regulating the Bioavailability of Vascular Endothelial Growth Factor A and the Expression of Nitric Oxide Synthase 1 and 3. EBioMedicine.

[53] Neunaber C, Catala-Lehnen P, Beil FT (2010). Increased trabecular bone formation in mice lacking the growth factor midkine. J Bone Miner Res.

[54] Erdogan S, Turkekul K, Dibirdik I (2018). Midkine downregulation increases the efficacy of quercetin on prostate cancer stem cell survival and migration through PI3K/AKT and MAPK/ERK pathway. Biomed Pharmacother.

[55] Ollauri-Ibáñez C, Núñez-Gómez E, Egido-Turrión C (2020). Continuous endoglin (CD105) overexpression disrupts angiogenesis and facilitates tumor cell metastasis. Angiogenesis.

[56] Mehta V, Fields L, Evans IM (2018). VEGF (vascular endothelial growth factor) induces NRP1 (neuropilin-1) cleavage via ADAMs (a disintegrin and metalloproteinase) 9 and 10 to generate novel carboxy-terminal NRP1 fragments that regulate angiogenic signaling. Arterioscler Thromb Vasc Biol.

[57] Kumagishi K, Nishida K, Yamaai T (2009). A disintegrin and metalloproteinase with thrombospondin motifs 9 (ADAMTS9) expression by chondrocytes during endochondral ossification. Arch Histol Cytol.

[58] Butler A, Hoffman P, Smibert P, Papalexi E, Satija R (2018). Integrating single-cell transcriptomic data across different conditions, technologies, and species. Nat Biotechnol.

[59] Shafer MER (2019). Cross-Species Analysis of Single-Cell Transcriptomic Data. Front Cell Dev Biol.

[60] Takao K, Miyakawa T (2015). Genomic responses in mouse models greatly mimic human inflammatory diseases. Proc Natl Acad Sci U S A.

[61] Junhee Seok, H (2013). Shaw Warren, Alex GC, et al. Genomic responses in mouse models poorly mimic human inflammatory diseases. Proc Natl Acad Sci U S A.

[62] Zhou W, Lin J, Zhao K (2019). Single-Cell Profiles and Clinically Useful Properties of Human Mesenchymal Stem Cells of Adipose and Bone Marrow Origin. Am J Sports Med.

[63] Chia LY, Walsh NC, Martin TJ, Sims NA (2015). Isolation and gene expression of haematopoietic-cell-free preparations of highly purified murine osteocytes. Bone.

[64] Zhong L, Yao L, Tower RJ (2020). Single cell transcriptomics identifies a unique adipose lineage cell population that regulates bone marrow environment. Elife.

[65] Yu W, Zhong L, Yao L (2021). Bone marrow adipogenic lineage precursors promote osteoclastogenesis in bone remodeling and pathologic bone loss. J Clin Invest.

[66] Liu Y, Beyer A, Aebersold R (2016). On the Dependency of Cellular Protein Levels on mRNA Abundance. Cell.

[67] Stoeckius M, Hafemeister C, Stephenson W (2017). Simultaneous epitope and transcriptome measurement in single cells. Nat Methods.

[68] Hu K, Olsen BR (2016). The roles of vascular endothelial growth factor in bone repair and regeneration. Vol. 91, Bone. Elsevier Inc.

[69] Hu K, Olsen BR (2016). Osteoblast-derived VEGF regulates osteoblast differentiation and bone formation during bone repair. J Clin Invest.

[70] Brotto M, Bonewald L (2015). Bone and muscle: Interactions beyond mechanical. Bone.

[71] Li G, Zhang L, Wang D (2019). Muscle-bone crosstalk and potential therapies for sarco-osteoporosis. J Cell Biochem.

[72] He C, He W, Hou J (2020). Bone and Muscle Crosstalk in Aging. Front Cell Dev Biol.

[73] Su X, Weng S, Peng D (2019). New insight into apolipoprotein A5 and the modulation of human adipose-derived mesenchymal stem cells adipogenesis. Curr Mol Med.

[74] Yiew NKH, Greenway C, Zarzour A (2019). Enhancer of zeste homolog 2 (EZH2) regulates adipocyte lipid metabolism independent of adipogenic differentiation: Role of apolipoprotein e. J Biol Chem.

[75] Han X, Zhou Z, Fei L (2020). Construction of a human cell landscape at single-cell level. Nature.

[76] Zilionis R, Engblom C, Pfirschke C (2019). Single-Cell Transcriptomics of Human and Mouse Lung Cancers Reveals Conserved Myeloid Populations across Individuals and Species. Immunity.

