# Supplementary material for: Single-cell RNA sequencing deconvolutes the in vivo heterogeneity of human bone marrow-derived mesenchymal stem cells
Source: Int J Biol Sci. 2021 Oct 11;17(15):4192–206. doi: 10.7150/ijbs.61950 (PMC8579438; doi:10.7150/ijbs.61950)
Supplement: Supplementary file 1 — Supplementary figures and table legends. [file ijbsv17p4192s1.pdf]

Supplementary Materials for

Single-cell RNA sequencing deconvolutes the in vivo heterogeneity of human bone marrow-derived mesenchymal stem cells

Zun Wang[§], Xiaohua Li[§], Junxiao Yang, Yun Gong, Huixi Zhang, Xiang Qiu, Ying Liu, Cui Zhou, Yu Chen, Jonathan Greenbaum, Liang Cheng, Yihe Hu, Jie Xie, Xucheng Yang, Yusheng Li, Martin R. Schiller, Yiping Chen, Lijun Tan, Si-Yuan Tang, Hui Shen, Hong-Mei Xiao*, Hong-Wen Deng*

§ These authors contributed equally to this work

***Corresponding author**

Hong-Wen Deng (hdeng2@tulane.edu)

Hong-Mei Xiao (hmxiao@csu.edu.cn)

Supplemental Figures

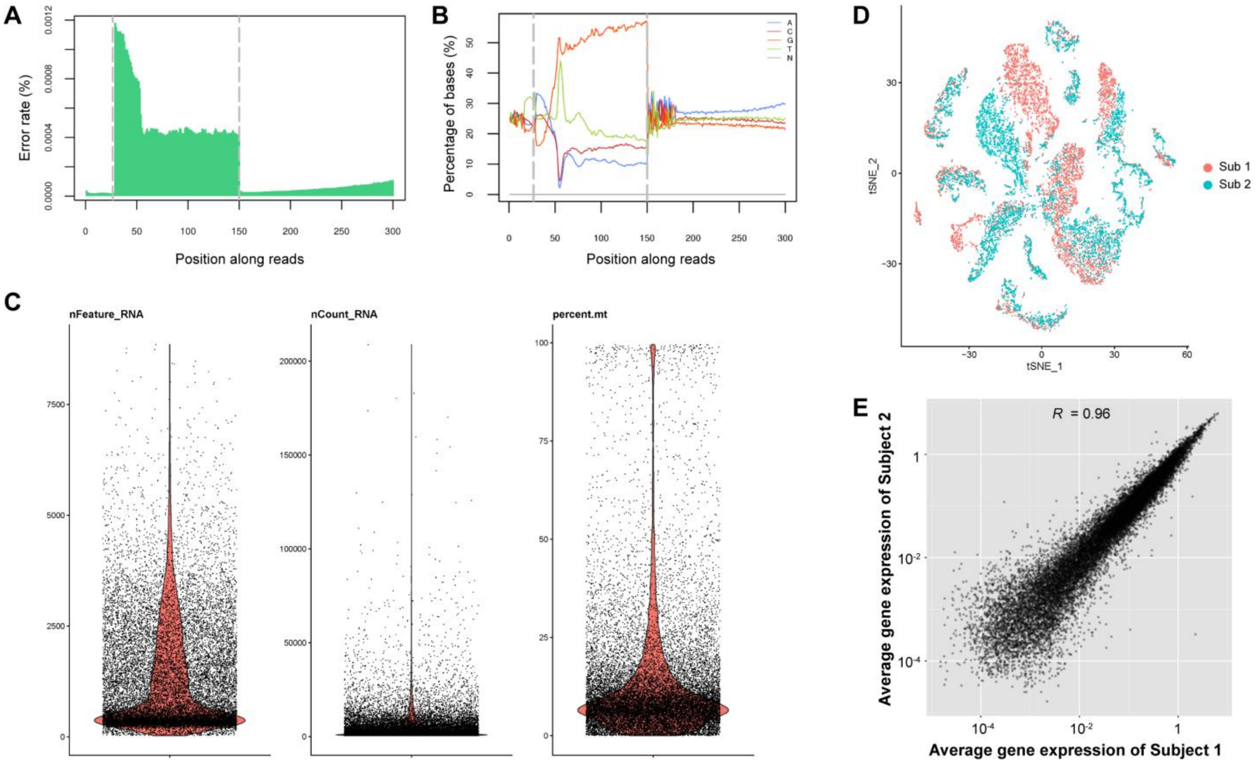

Figure S1. Quality control for scRNA-seq data. (A) Average error rate distribution of base pair. x-axis represents position of base pair along reads while y-axis represents the error rate. Dashed line at 150 bp separates Read1 and Read2 (barcode and RNA sequence, representative). The average error rate of Read2 (RNA sequence) was less than 1×10^{-4}. **(B)** Base content distribution. x-axis represents position of base pair along reads while y-axis represents the proportion of different nucleotides (A, T, G, C, N stands for uncertain). Dashed line at 150 bp separates Read1 and Read2. All the bases were determined to A, T, G, or C with no undetermined bases, and difference between A and T, or G and C is less than 10% in any position. Combined with (A), it suggests a good quality of raw sequencing data. **(C)** Distribution of the number of detected genes (features), RNA molecules, and proportion of mitochondrial genes (left to right). Mean Reads per Cell: 5,458; Mean Genes per Cell: 1,339. In less than 20% of cells that proportion of mitochondrial genes is over 20%, therefore in the majority of the cells, most of the detected reads and genes are from the genome. **(D)** t-SNE dimension reduction, colored by different subjects (sub1, osteoporosis, red, n = 7,498; sub2, osteopenia, blue n = 6,996). **(E)** Correlation of gene expression between two subjects. Each dot represents an

individual gene. Axis measure the average gene expression level in the indicated subject (axis is log-scaled). Correlation was tested by Pearson correlation coefficient ($R = 0.96$, $p < 0.01$).

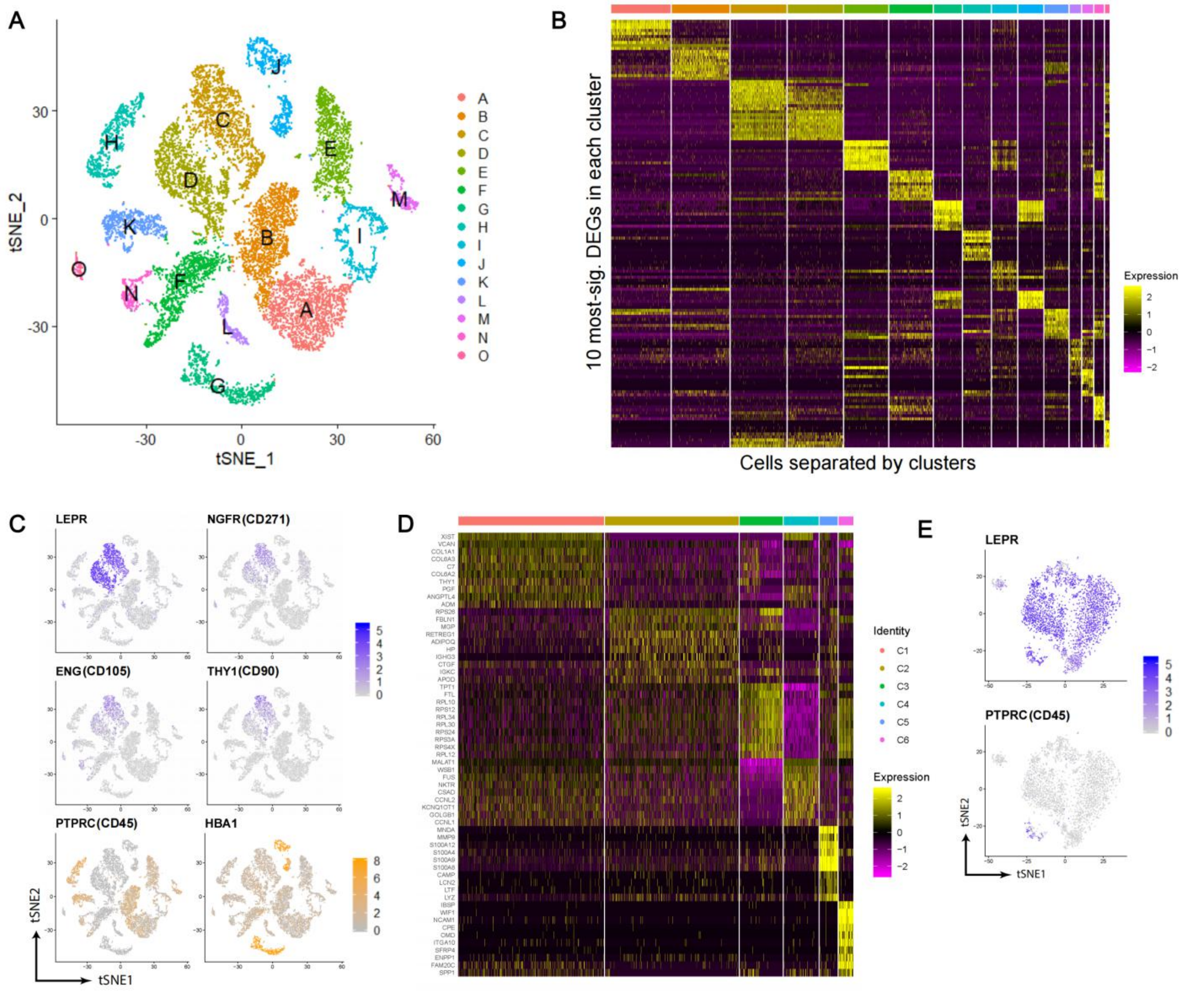

Figure S2. Cluster and marker gene identification for scRNA-seq data. **(A)** t-SNE dimension reduction, colored by different clusters. **(B)** Gene expression profile of $CD271^{+}$ BM-MNCs, based on the relative gene expression level of top 10 most-significant markers for each cluster. **(C)** Gene expression of known BM-MSC markers, embedded on t-SNE dimension reduction map, and colored by gene expression level. **(D)** Gene signature of BM-MSCs, based on the relative gene expression level of top 10 most-significant DEGs for each cluster (z-score). **(E)** Gene expression of LEPR and PTPRC (CD45) in BM-MSCs, embedded on t-SNE dimension reduction map, and colored by gene expression level (logFC).

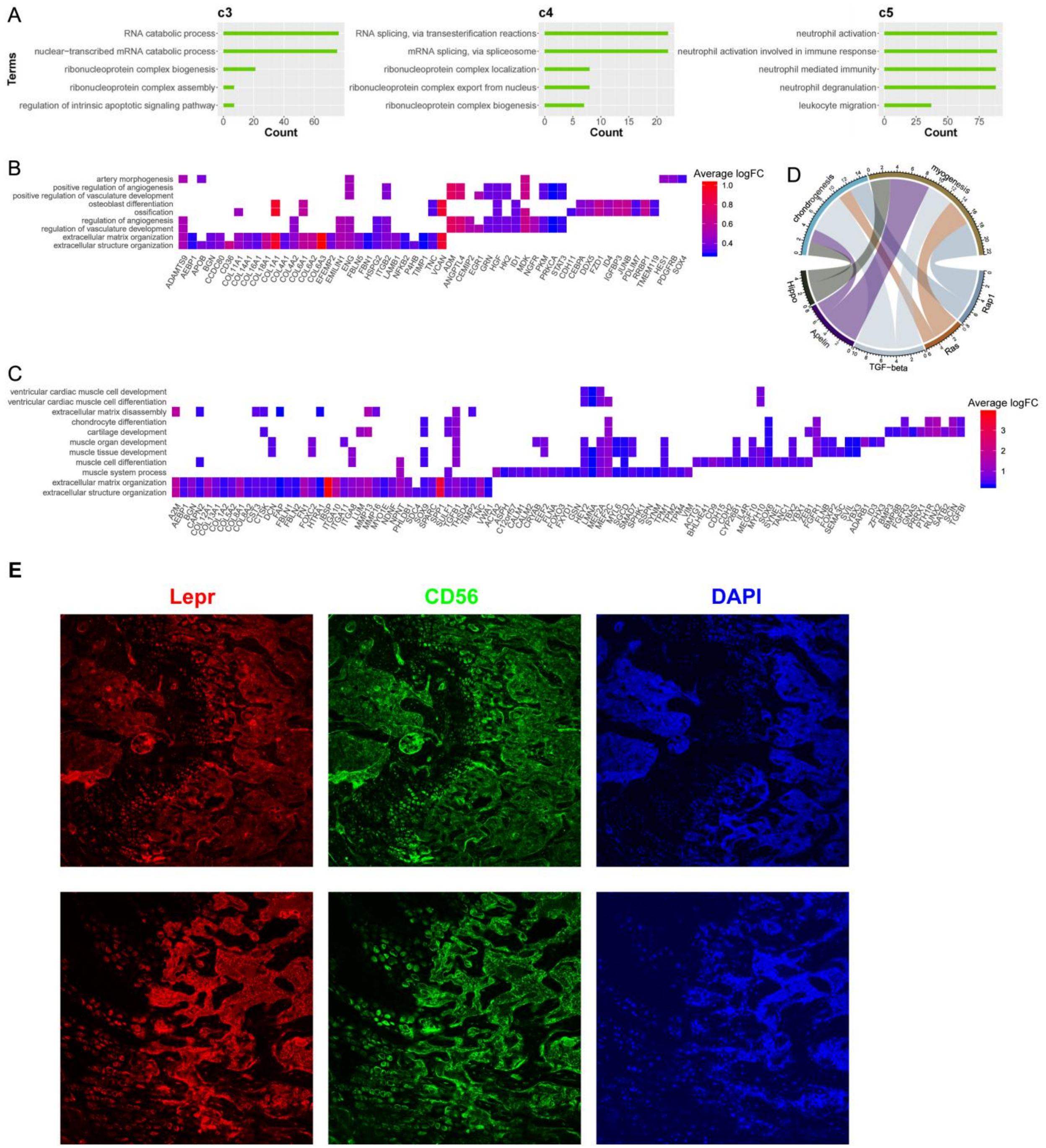

Figure S3. GO enrichment for BM-MSC. (A) Enriched GO terms in cluster 3 to 5 (left, middle, right, respectively). Bar chart shows the number of enriched genes in each term. **(B-C)** Gene expression pattern in enriched pathways for osteoblast **(B)** and chondrocyte **(C)** precursors. Squares showing enrich DEGs in the corresponding terms (rows). Color indicating the gene expression level (average logFC). **(D)** Common genes shared between biological processes and

pathways. Width of curves connecting different terms indicating the relative proportion of the shared genes. **(E)** Individual channel for Fig 4b. Top: low magnification; Bottom: high magnification.

A

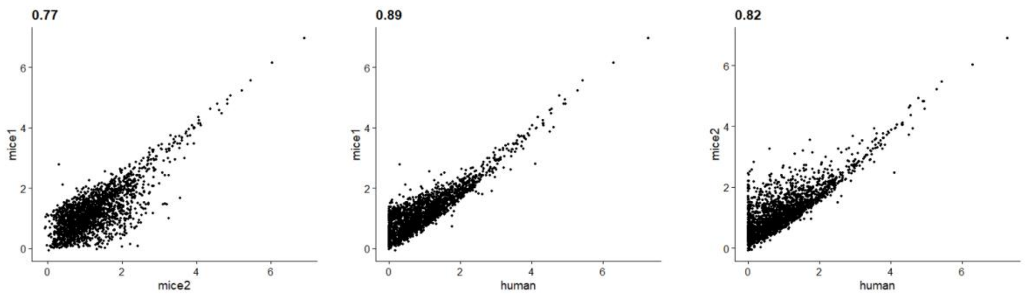

B

Species	CD markers
Human	CD317, CD36, CD63, CD249, CD39, CD91, CD10, CD106
Mice	CD148, CD108, CD20, CD7, CD272

C

	Human	Mice
**Osteogenesis**	IBSP, IFITM1, SFRP1, SMAD5, COL6A1, CDH11, JUNB, ID4, CSF1, SATB2, H3F3A, SPARC, RPL38, IGFBP5, MGP, SRGN, TPM4, CD36, VCAM1, LRP1, EFEMP2, CST3, ADAMTS9, EMILIN1, LUM, THBS1, TIMP1, EFEMP1, SOX4, GLG1, FOXP1, HMOX1, ADM, MAP1LC3B, FOS, GLUL, ZFP36, TMBIM6, COX4I1	FZD9, ASPN, CTHRC1, P2RX7, ITGA11, SEMA7A, SBNO2, VWF, TSG101, SERPINE1, ICAM5, COL4A2, ADAMTS12, TBX3, FGFRL1, SERPINC1, GCLC, UBASH3B
**Adipogenesis**	CD36, ESYT2, LRP1, THBS1, ADM, KPNB1, EDF1, ZFP36, SFRP1, ID4, HIP1, RFTN1, CSF1, UBB, HMOX1, GLUL, SLC44A2, DDHD1, CYGB, S1PR3, UNC5B	SMPD2, IP6K3, SMG1, PIGT, MBOAT1, P2RX7, ST8SIA4, ABCC1, ZDHHC16, PDP2, UNC119

Figure S4. Comparison of gene expression profile between hBM-MSCs and mBM-MSCs. (**A**) Correlations of gene expression among different BM-MSC datasets after CCA integration. Each dot represents an individual gene. The average gene expression level (logFC) are plotted for each subject. Correlations were measured by Pearson correlation coefficients (R) ($p < 0.01$). (**B**) Comparison of differentially expressed CD markers between human and mice BM-MSCs. (**C**) Table comparing enriched genes in osteogenesis- and adipogenesis- related terms between human and mice BM-MSCs.

Supplemental Tables

Table S1. Differential gene expression analysis.
Sheet 1: Cluster-specific DEGs of each cluster in $CD271^{+}$ BM-MSCs. Sheet 2: DEGs in $LEPR^{hi}CD45^{low}$ BM-MSCs when comparing with other $CD45^{hi}$ BM-MNCs. Sheet 3: Cluster-specific DEGs for BM-MSC clusters. Sheet 4: Relative gene expression between hBM-MSCs and mBM-MSCs. Average log-transformed fold change (logFC) was calculated on averaging the expression value of all single cells in each cluster against other clusters using Wilcoxon rank-sum test.

Table S2. Enriched GO and KEGG terms for BM-MSCs.
Sheets 1-2: Enriched GO and KEGG terms of cluster-specific DEGs six identified clusters in BM-MSCs, respectively. Sheets 3-4, Enriched GO and KEGG terms of DEGs between hBM-MSCs and mBM-MSCs, respectively.

Table S3. Expression pattern of enriched genes in related biological processes. Sheets 1-2: Enriched genes in osteoblast and chondrocyte precursors, respectively.
